# Proteomic Profiling of IgG1 Producing CHO Cells Using LC/LC-SPS-MS^3^: The Effects of Bioprocessing Conditions on Productivity and Product Quality

**DOI:** 10.3389/fbioe.2021.569045

**Published:** 2021-04-09

**Authors:** Lisa Strasser, Amy Farrell, Jenny T. C. Ho, Kai Scheffler, Ken Cook, Patrick Pankert, Peter Mowlds, Rosa Viner, Barry L. Karger, Jonathan Bones

**Affiliations:** ^1^Characterization and Comparability Laboratory, National Institute for Bioprocessing Research and Training, Dublin, Ireland; ^2^Thermo Fisher Scientific, Hemel Hempstead, United Kingdom; ^3^Thermo Fisher Scientific, Germering, Germany; ^4^Thermo Fisher Scientific, Dreieich, Germany; ^5^Thermo Fisher Scientific, San Jose, CA, United States; ^6^Barnett Institute of Chemical and Biological Analysis, Northeastern University, Boston, MA, United States; ^7^School of Chemical and Bioprocess Engineering, University College Dublin, Dublin, Ireland

**Keywords:** CHO cells, TMT, proteomic profiling, bioprocess monitoring, protein characterization, SPS-MS^3^, monoclonal antibody

## Abstract

The biopharmaceutical market is dominated by monoclonal antibodies, the majority of which are produced in Chinese hamster ovary (CHO) cell lines. Intense cell engineering, in combination with optimization of various process parameters results in increasing product titers. To enable further improvements in manufacturing processes, detailed information about how certain parameters affect cellular mechanisms in the production cells, and thereby also the expressed drug substance, is required. Therefore, in this study the effects of commonly applied changes in bioprocessing parameters on an anti-IL8 IgG1 producing CHO DP-12 cell line were investigated on the level of host cell proteome expression combined with product quality assessment of the expressed IgG1 monoclonal antibody. Applying shifts in temperature, pH and dissolved oxygen concentration, respectively, resulted in altered productivity and product quality. Furthermore, analysis of the cells using two-dimensional liquid chromatography-mass spectrometry employing tandem mass tag based isotopic quantitation and synchronous precursor selection-MS^3^ detection revealed substantial changes in the protein expression profiles of CHO cells. Pathway analysis indicated that applied bioprocessing conditions resulted in differential activation of oxidative phosphorylation. Additionally, activation of ERK5 and TNFR1 signaling suggested an affected cell cycle. Moreover, in-depth product characterization by means of charge variant analysis, peptide mapping, as well as structural and functional analysis, revealed posttranslational and structural changes in the expressed drug substance. Taken together, the present study allows the conclusion that, in anti-IL8 IgG1 producing CHO DP-12 cells, an improved energy metabolism achieved by lowering the cell culture pH is favorable when aiming towards high antibody production rates while maintaining product quality.

## Introduction

With an increasing number of approvals every year, the biopharmaceutical market is dominated by monoclonal antibodies (mAbs) which are most commonly produced in Chinese hamster ovary (CHO) cells (Kaplon and Reichert, 2018; Walsh, 2018). Consequently, CHO cells have been actively engineered over the last decade in order to express high levels of a wide range of mAbs and other therapeutic biomolecules. Various strategies have been applied to enable optimized CHO cell bioprocessing, aiming not only for an increased specific productivity but also for a higher cell viability while reducing the amount of undesired by-products (Kunert and Reinhart, 2016). Frequently used approaches include shifts in cell culture parameters like temperature and pH as well as the addition of certain media supplements or the use of controlled feeding strategies (Trummer et al., 2006; Fan et al., 2015; Vergara et al., 2018). Various studies have shown that a temperature shift from 37 to 32°C drastically increases recombinant protein production, potentially due to improved cell viability (Yoon et al., 2004; Ahn et al., 2008). However, it was also shown that an improved energy metabolism under hyperosmotic conditions favors specific productivity of CHO cells (Lee et al., 2003). While most of these studies focused on the effects of one factor at a time, more systemic approaches such as quality by design and design of experiments have been used to study the link between applied parameters whilst further pushing the limits of recombinant protein production (Looby et al., 2011; Nagashima et al., 2013; Weng et al., 2020).

Nevertheless, there is still limited knowledge about of how these process alterations affect fundamental biological processes within the production cells and how these alterations ultimately relate to quality attributes such as, e.g., charge variants which could alter product efficacy and safety (Torkashvand and Vaziri, 2017; Papathanasiou and Kontoravdi, 2020). For this reason ‘omics techniques have been applied to explore CHO cells under various conditions in order to provide a foundation for knowledge-driven improvements of manufacturing processes (Baycin-Hizal et al., 2012; Farrell et al., 2014; Heffner et al., 2017; Stolfa et al., 2018; Ali et al., 2019, 2020; Lakshmanan et al., 2019). More accurate and complete genome references (Rupp et al., 2018) in combination with technological improvements in analytical instrumentation (Williamson et al., 2016) now enable proteomic profiling of CHO cells to a depth and accuracy that was previously not possible.

Since the early days of CHO cell proteomics, quantification methods have steadily evolved (Bachi and Bonaldi, 2008; Bakalarski and Kirkpatrick, 2016). Isobaric labeling-based techniques in particular have proven powerful as they can provide high throughput while avoiding run-to-run variations (Thompson et al., 2003; Piehowski et al., 2013; Pappireddi et al., 2019). Yet, it was shown that precursor ion co-isolation can lead to ratio compression and thus an overestimation of protein expression (Karp et al., 2010). Therefore, state-of-the-art Orbitrap tribrid mass spectrometers facilitate synchronous precursor selection (SPS) prior to MS^3^ (SPS-MS^3^) detection. In this method, MS^2^ fragment ions are isolated using SPS followed by an additional fragmentation step and MS^3^ detection which reduces co-isolation and thus enables more accurate quantitation when using labeling reagents such as tandem mass tags (TMT) or other isobaric tags like iTRAQ for relative quantitation (Ting et al., 2011; McAlister et al., 2014).

In this study, an anti-IL8 IgG1 producing CHO DP-12 cell line was used to investigate the effects of alterations in bioprocessing parameters such as temperature, pH, or dissolved oxygen (DO) concentration on the host cell proteome. Even though the mAb under investigation is not a therapeutic product, as a CHO derived IgG1 it is similar to therapeutic biomolecules undergoing biopharmaceutical production and has been widely used as a model (Pezzini et al., 2011; Beckmann et al., 2012; Perez-Rodriguez et al., 2020). By using offline high pH reversed phase liquid chromatography (LC) fractionation, followed by low pH reversed phase nano LC-SPS-MS^3^ analysis employing TMT quantitation, more than 9,200 proteins were identified and over 6,600 proteins were quantified across all samples. This in-depth proteome analysis revealed significant impact of applied conditions on cell metabolism. To gain knowledge of how these cellular processes affect product quality, comprehensive characterization of the drug substance was performed.

To the best of our knowledge, this is the first time altered bioprocessing conditions have been studied not only on the level of host cell proteome but also correlated with levels of expressed product quality. The findings in this study underline the importance of combining multiple levels of analysis to obtain a holistic view on how process parameters affect the expression system and consequent product quality profile.

## Materials and Methods

### CHO DP-12 Cell Culture Using Disposable Single Use Bioreactors

Bioprocessing studies were performed using Sartorius Stedim Cultibag systems equipped with 2.0 L Optical Cultibag single use bioreactors (Sartorius Stedim Biotech GmbH, Göttingen, Germany). Bioreactors were placed on temperature-controlled rocker stations and fitted with gas lines, DO and pH sensors and a base addition line. The culture was initially maintained at 37.0°C and a pH of 7.0 by the automated addition of 1.0 M sodium hydroxide or CO_2_ gas, while DO levels were kept at 85.0%. Three times three bioreactor cultivations were performed in parallel at a starting volume of 1.0 L using EX-CELL 325 PF CHO media containing 10.0 mg/L recombinant human Insulin, 4.0 mM L-Glutamine, 1.0 μM methotrexate (Sigma Aldrich, Wicklow, Ireland) and 20,000 units of Penicillin-Streptomycin (Fisher Scientific, Dublin, Ireland). CHO DP-12 [CHO DP-12, clone#1934 aIL8.92 NB 28605/14] (ATCC CRL-12445) cells, purchased from LGC standards (Middlesex, United Kingdom), which were adapted to serum-free suspension culture were inoculated at a starting viable cell density of 3.0 × 10^5^ cells/mL. As the cell cultures began to enter the stationary phase of cell growth, one parameter in two of the bioreactors was changed as shown in Table 1. Cultures were allowed to proceed for 48 h at altered conditions after which time they were harvested by transferring each culture into multiple falcon tubes for centrifugation at 125 × *g* for 5 min at 4°C. Supernatant and cell pellets were stored at −80°C until further processing.

**TABLE 1:** Bioprocessing conditions used to prepare CHO DP-12 cell cultures in Sartorius Cultibag Disposable Bioreactors.

Applied bioreactor settings once cells entered stationary phase (9 bioreactors in total)			
Parameter	High	Standard	Low
Temperature	High temperature: 39.5°C, pH 7.0, 85% DO	Standard 1: 37.0°C, pH 7.0, 85% DO	Low temperature: 32.0°C, pH 7.0, 85% DO
pH	High pH: 37.0°C, pH 7.2, 85% DO	Standard 2: 37.0°C, pH 7.0, 85% DO	Low pH: 37.0°C, pH 6.8, 85% DO
DO	High DO: 37.0°C, pH 7.0, 110% DO	Standard 3: 37.0°C, pH 7.0, 85% DO	Low DO: 37.0°C, pH 7.0, 60% DO

*In total, 9 bioreactors were used resulting in 3 biological replicates for standard conditions. After day 5, cells were cultured under altered conditions for 48 h before harvest.*

### Determination of Nutrient and Metabolite Concentration, Cell Count and Cell Viability

**FIGURE 2:**
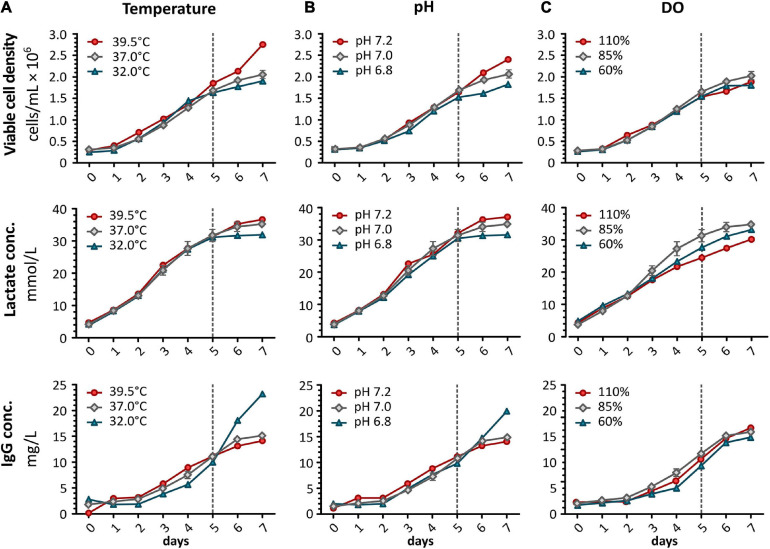
For bioprocess monitoring of CHO DP-12 batch cultures, viable cell density was determined daily using the trypan blue exclusion method. Additionally, lactate concentration and IgG production were analyzed using a Cedex Bio Analyzer (Roche). Shown are results after shifting **(A)** temperature, **(B)** pH, or **(C)** DO concentration. Red shows an increase whereas blue indicates a reduction of the correlating parameter. Results obtained from standard conditions are shown in gray (mean ± standard deviation, *n* = 3). Dashed line indicates time point (day 5) for alterations of process parameters.

Sampling for measurements of cell density and metabolites was performed daily by removing a small aliquot from the bioreactor. Cell count was performed using the trypan blue exclusion method (Strober, 2001) and a hemocytometer. For determination of nutrient and metabolite concentration, 1.0 mL of each culture was centrifuged (5,000 × *g*, 10 min) to remove cellular material before photometric analysis with a Cedex Bio Analyzer (Roche Diagnostics Deutschland GmbH, Mannheim, Germany) according to the manufacturer’s instructions. Thereby, concentration of IgG, glucose, glutamine, lactate, ammonia, and glutamate as well as LDH activity were determined, respectively (data partially shown in Figure 2).

### Proteomic Profiling of CHO Cells Using Two-Dimensional High pH-Low pH LC-MS Analysis

#### Sample Preparation

**FIGURE 1:**
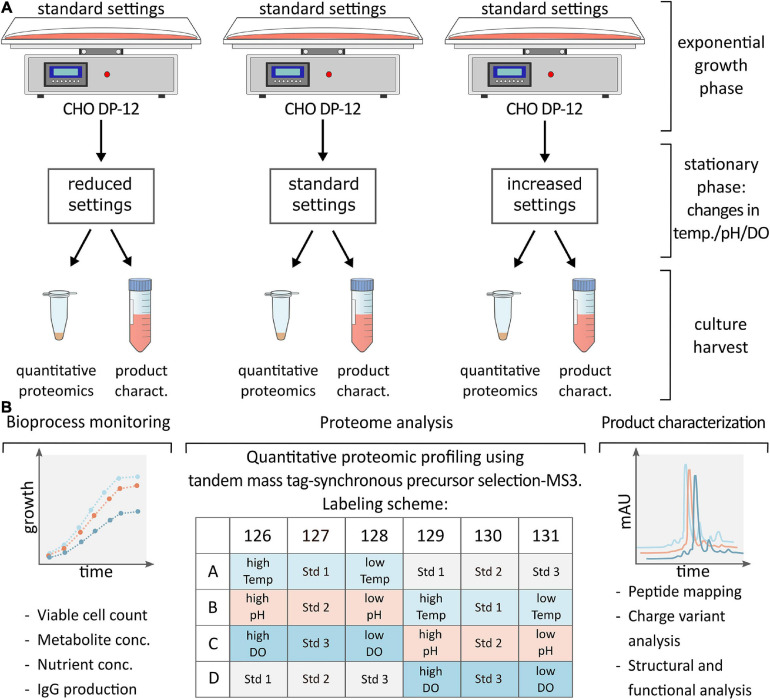
**(A)** CHO DP-12 cells were grown under standard conditions until cells entered stationary phase (day 5) then process parameters were changed as shown followed by cell harvest and supernatant collection after 48 h. **(B)** Schematic overview of analysis following cell harvest. Bioprocess monitoring was carried out on a daily basis to assess viable cell density, metabolite or nutrient concentration and IgG production rate. Following batch culture, cells were lysed, and samples were prepared for quantitative proteomic profiling applying TMT labeling as indicated. Complementary product characterization was done to detect sequence variants, modifications, and structural changes.

Cell pellets were thawed on ice followed by three washes using 1.0 mL of Dulbecco’s phosphate buffered saline (PBS). Afterwards, cells were reconstituted at a concentration of 1.0 × 10^7^ cells/mL in 8.0 M urea in 100.0 mM Tris buffer, pH 8.0 (Sigma Aldrich). Cells were lysed *via* sonication for 30 s using a Fisherbrand Model 50 Sonic Dismembrator (Fisher Scientific) set to 20.0 kHz followed by centrifugation at 16,000 × *g* for 10 min. Protein concentration of the supernatant was determined using a Bradford protein assay (Sigma Aldrich). Duplicates of generated CHO cell lysates (*n* = 2) were then used for proteomic sample preparation using a filter-aided sample preparation (FASP) protocol (Wisniewski et al., 2009). Briefly, protein aliquots of 100 μg per sample were added to 10 kDa molecular weight cutoff (MWCO) filters followed by reduction using 5.0 mM dithiothreitol (DTT) at 25°C for 30.0 min and alkylation with 15.0 mM iodoacetamide (IAA) for 30 min in the dark. Following buffer exchange into 100.0 mM triethylammonium bicarbonate (TEAB, Sigma Aldrich), enzymatic digestion was performed overnight with trypsin (MyBio Ltd., Kilkenny, Ireland) at 37°C using a 1:50 (m/m, enzyme:protein) ratio. Subsequently, peptides were retrieved by centrifugation at 14,000 × *g* for 10 min before labeling with a TMTsixplex Isobaric Label Reagent Set (Thermo Fisher, Rockford, IL, United States) according to the manufacturer’s instructions. After labeling as per the experimental design shown in Figure 1, samples were pooled and reduced to dryness *via* vacuum centrifugation using Thermo Scientific’s Savant SPD111V vacuum concentrator.

#### High pH Fractionation

First dimensional separation was done using an Acquity H-Class UPLC instrument (Waters, Dublin, Ireland) with UV detection at 214 nm. Thus, dried peptide samples were reconstituted in 500.0 μL of 0.10% (v/v) formic acid (FA) in water (LC-MS Optima, Fisher Scientific) prior to loading onto an Acquity UPLC BEH 130 C18, 1.7 μm, 2.1 × 150 mm (Waters) analytical column. Separation of peptides was performed at pH 10.0 using a gradient of 10.0 mM ammonium formate in water (A) and 10.0 mM ammonium formate in 95.0% (v/v) acetonitrile (ACN; B; Fisher Scientific). Gradient conditions were as follows: 3.0% B initially for 2 min, increased to 10.0% B in 2 min with a further increase to 45.0% B over 33 min followed by a final increase to 100% B in 2 min with a 2 min isocratic hold. Initial conditions were restored and held for 10 min for column re-equilibration. The column temperature was maintained at 40.0°C, and the flow rate was 200.0 μL/min. Eluting peptides were fractionated every minute and subsequently recombined to 10 sample fractions of equal protein concentration. Finally, sample fractions were evaporated to dryness *via* vacuum centrifugation using Thermo Scientific’s Savant SPD111V vacuum concentrator.

#### Reversed Phase Nano-LC-SPS-MS^3^ Analysis

All spectra were acquired using a Thermo Scientific Orbitrap Fusion Lumos Tribrid mass spectrometer (Thermo Fisher Scientific, San Jose, United States) coupled to a Thermo Scientific UltiMate 3000 RSLCnano HPLC system by means of a Thermo Scientific EASY-Spray source (Thermo Fisher Scientific, Germering, Germany). Sample fractions were reconstituted in 0.10% (v/v) FA in water to a final peptide concentration of 1.0 μg/μL. 1 μg per sample was loaded onto a Thermo Scientific EASY-Spray Acclaim PepMap 100, 75 μm × 50 cm column (Thermo Fisher Scientific, Sunnyvale, CA, United States) maintained at 40.0°C at a flow rate of 300.0 nL/min. Separation was achieved using a gradient of 0.10% (v/v) FA in water (A) and 0.10% (v/v) FA in ACN (B; LC-MS optima, Fisher Scientific). Gradient conditions were as follows: 5.0% B initially for 5 min, increased to 25.0% B in 85 min with a further increase to 60.0% B over 20 min followed by a final increase to 90.0% B in 5 min with a 5 min isocratic hold. Initial conditions were restored and held for 30 min for column re-equilibration.

The mass spectrometer was operated in the positive ion mode, and a spray voltage of 1.8 kV was applied. The instrument was operated in data dependent acquisition top speed mode where one cycle consisted of a MS^1^ survey scan acquired in the Orbitrap covering a scan range of *m/z* 400 to 1,500 at a resolution setting of 120,000 (at *m/z* = 200) with an automatic gain control (AGC) target of 4 × 10^5^ and a maximum injection time (IT) of 50 ms. The most intense precursor ions with a charge state between 2 and 7 were isolated in the quadrupole using an AGC target of 1 × 10^4^ and an isolation window of 0.7 *m/z*. The maximum IT for MS^2^ scans was set to 50 ms. Following isolation, precursors were fragmented using CID fragmentation at 35% normalized collision energy (NCE), and the resulting fragment ions were detected in the ion trap mass analyzer. SPS was enabled to select fragment ions for an MS^3^ scan in the Orbitrap at a resolution setting of 60,000 (at *m/z* = 200). Using an isolation window of 2 *m/z* and a mass range of *m/z* 100 to 500, the ten most intense ions were further fragmented using HCD fragmentation with 65% collision energy to ensure maximal TMT reporter ion yield. The AGC target for the MS^3^ scans was set to 1 × 10^5^ with a maximum IT of 105 ms.

#### Data Analysis

Raw data was processed using Thermo Scientific Proteome Discoverer software version 2.3 and deposited to the ProteomeXchange Consortium via the PRIDE partner repository (Vizcaino et al., 2016) with the dataset identifier PXD017779. For protein identification, MS^2^ spectra were searched against a *Cricetulus griseus* database (Uniprot downloaded 27th of May 2019) using the Sequest HT search engine. A mass tolerance of 10 ppm for precursor ions and 0.6 Da for fragment ions was allowed. Additionally, a maximum of 2 missed cleavages was accepted. Search criteria also included carbamidomethylation of cysteines and TMTsixplex labeled peptide amino terminus and lysine residues (+229.263 Da) as static modification whereas oxidation and *N*-terminal acetylation were used as dynamic modifications. Normalization of TMT reporter intensity values was performed based on total peptide amounts.

Following identification and quantification in Proteome Discoverer software, statistical analysis was undertaken using the Perseus software (version 1.6.6). Significance of log2(x)-ratios (*n* = 2) was determined using Volcano plots employing a two-sided *t*-test with a false discovery rate (FDR) of 0.05 and a variance parameter (S0) of 0.03. For biological interpretation, a gene ontology (GO) enrichment analysis was done using a Fisher’s exact test (*p* ≤ 0.05). Additionally, gene names were imported into QIAGEN’s Ingenuity Pathway Analysis software (IPA, QIAGEN Redwood City, www.ingenuity.com) to reveal pathway activation in response to altered bioprocessing conditions.

### Characterization of Monoclonal Anti-IL8 Antibody

#### Purification of Anti-IL8 From CHO DP-12

Media containing anti-IL8 monoclonal antibody derived from CHO DP-12 culture was thawed before clarification *via* sequential filtration through 0.45 μm and 0.20 μm syringe filters (VWR, Dublin, Ireland). Clarified media was then passed through a HiTrap Protein A column (GE Healthcare, Dublin, Ireland) at a flow rate of 1.0 mL/min. Using PBS, the column was washed to remove unretained material followed by elution of the mAb by 100.0 mM L-arginine (Sigma Aldrich) at pH 3.5. The pH of the eluate was neutralized (pH 7.0–7.5) by adding 0.50 M Tris buffer followed by a buffer exchange into PBS using Amicon Ultra centrifugal units with a 10 kDa MWCO membrane (Sigma Aldrich). The IgG content was determined using a Bradford protein assay; subsequently, aliquots containing 100 μg of protein were stored at −30°C pending further analysis.

#### Cation Exchange Chromatography

Charge variant analysis (CVA) of intact mAb was performed using a Thermo Scientific Vanquish Flex UHPLC system equipped with a diode array detector at 280 nm (Thermo Fisher Scientific, Germering, Germany). Separation based on a pH gradient was achieved using a Thermo Scientific MAbPac SCX-10 RS, 5 μm, 2.1 × 50 mm analytical column with CX-1 pH gradient buffer A, pH 5.6 and CX-1 pH gradient buffer B, pH 10.2 (Thermo Scientific, Sunnyvale, CA, United States). Elution of sample peaks was performed using a linear gradient of 35–55% B in 30 min at a temperature of 25°C and a flow rate of 0.50 mL/min. Thermo Scientific Chromeleon CDS 7.2 was used for data acquisition and analysis.

#### Peptide Mapping

Peptide mapping was performed as published previously (Farrell et al., 2018). Briefly, samples were prepared applying a FASP protocol as described above with some minor changes. 50 μg of protein per sample were loaded onto 10 kDa MWCO filters followed by reduction using 5.0 mM DTT at 65°C for 30 min and alkylation using IAA. Samples were then buffer exchanged into 50.0 mM ammonium bicarbonate (Sigma Aldrich) followed by tryptic digestion overnight. Afterward, peptides were eluted by centrifugation and evaporated to dryness prior to further analysis.

Separation of mAb peptides was performed with a Thermo Scientific Vanquish Horizon UHPLC System (Thermo Fisher Scientific, Germering, Germany). Tryptic peptides were then reconstituted in 0.10% (v/v) FA in water and loaded onto a Thermo Scientific Acclaim 120 C18, 5 μm, 2.1 × 250 mm analytical column (Thermo Fisher Scientific, Sunnyvale, CA, United States). A binary gradient of 0.10% (v/v) FA in water (A) and 0.10% (v/v) FA in ACN (B) was used employing the following conditions: 2.0 to 30.0% B in 40 min with a further increase to 80.0% B over 3 min followed by a 3.5 min hold. Initial conditions were restored in 0.5 min, and the column was re-equilibrated for 18 min. The column temperature was kept at 60°C at a flow rate of 250.0 μL/min.

The UHPLC system was on-line hyphenated to a Thermo Scientific Q Exactive Plus Hybrid Quadrupole-Orbitrap mass spectrometer using a Thermo Scientific Ion Max API source equipped with a heated electrospray ionization probe (Thermo Fisher Scientific, Bremen, Germany). The mass spectrometer was operated in the positive ion mode, and a spray voltage of 3.5 kV was applied. The capillary temperature was set to 320°C, and sheath and auxiliary gas flows were set to 20 and 8 arbitrary units, respectively. Data was acquired in a data dependent mode. Following a full scan at a scan range of *m/z* 200–2,000 with a resolution setting of 70,000 (at *m/z* = 200), the 5 most intense precursors were selected for fragmentation. The AGC target for full scans was 3 × 10^6^ with a maximum IT of 100 ms. Following isolation of precursors in the quadrupole using an isolation window of 2.0 *m/z*, ions were fragmented by HCD fragmentation at 28% NCE followed by a MS^2^ scan in the Orbitrap with a resolution setting of 17,500 (at *m/z* = 200). For MS^2^ scans, the AGC target setting was 1 × 10^5^ and a maximum IT of 200 ms.

Resulting data was processed using Thermo Scientific BioPharma Finder version 1.0.76.10 with a mass accuracy of 5 ppm and a maximum of one modification per peptide. *N* terminal glutamine to pyro-glutamate conversion, *C*-terminal lysine clipping, side chain carbamidomethylation, and carboxymethylation, deamidation (N, Q) and oxidation (M, W) were allowed as variable modifications. Data were searched against a FASTA file composed of the anti-IL8 IgG1 sequence obtained from United States Patent 6117980 (Gonzalez et al., 2000).

#### Hydrogen-Deuterium Exchange Mass Spectrometry

Hydrogen-Deuterium Exchange Mass Spectrometry (HDX-MS) was performed using a nanoAcquity UPLC M-class system with a LEAP Hydrogen-Deuterium Exchange (HDX) automation manager coupled to a SYNAPT G2 HDMS QToF (Waters, Millford, MA, United States). Purified IgG1 samples were diluted to a concentration of 5.0 mg/mL in 10.0 mM potassium phosphate (Sigma Aldrich), pH 7.0. For labeling, 4.80 μL of mAb was diluted with 20-fold 10.0 mM potassium phosphate in deuterium oxide (D_2_O; Sigma Aldrich), pH 6.6 at room temperature (20–25°) and incubated for 20 s, 1 min, 2 min, 10 min, 30 min, 1 h, 2 h, and 4 h, respectively. The reaction was quenched by adding 50.0 mM potassium phosphate, 2.0 M guanidine-HCl, 200.0 mM tris(2-carboxyethyl)phosphine (TCEP; Sigma Aldrich), pH 2.30 before on-line pepsin digestion using an Enzymate Pepsin, 300Å, 5 μm, 2.1 × 30 mm column (Waters) at 20.0°C.

After digestion, all chromatographic steps were performed at 0°C. First, eluting peptides were trapped on an Acquity UPLC BEH C18, 1.7 μm, 2.1 × 5 mm VanGuard pre-column (Waters) using 0.10% (v/v) FA in water (A) and 5.0% of 0.10% (v/v) FA in ACN (B) for 3.0 min at 200.0 μL/min. Separation was achieved using an Acquity UPLC BEH C18, 1.7 μm, 1.0 × 100 mm column (Waters) by applying a linear gradient of 5.0 to 35.0% B in 6 min followed by an increase to 95.0% B in 1 min and a 3 min isocratic hold at a flow rate of 40.0 μL/min. This was followed by three more washes before the column was re-equilibrated with 5.0% B. The chromatographic system was directly coupled to the QToF mass spectrometer by means of an electrospray ionization source using a spray voltage of 3.0 kV. The mass spectrometer was operated in the positive ion data independent resolution mode with 0.3 s sequential low and high energy scans in the range of 50 to 2,000 Da. A collision energy ramp from 15 V to 35 V was used for the high energy function. [Glu^1^]-fibrinopeptide (*m/z* 785.8426, *z* = 2, Sigma Aldrich) was used as lockmass, recorded in 30 s intervals. Non-deuterated control experiments were used to identify all peptides produced during enzymatic digestion.

Resulting raw data for unlabeled samples was processed using ProteinLynx Global Server software (PLGS, version 3.0.1, Waters) for identification of sample peptides. Workflow parameters included automatic peptide and fragment tolerances with lockmass correction and a minimum number of 2 fragment ion matches per peptide as well as a minimum 5 fragment ion matches per protein and ≥1 unique peptides per protein. Carbamidomethylation of cysteines was used as fixed modification while oxidation (M) and deamidation (N, Q) were set as variable modifications. Using a 1% FDR and a maximum of 1 missed cleavage, data was searched against a FASTA file composed of the anti-IL8 mAb sequence (Gonzalez et al., 2000).

All HDX-MS data was processed using DynamX HDX data analysis software version 3.0.0 (Waters) with additional manual verification of the raw spectra. A minimum sequence coverage of 70% and average redundancy of <5 was required for all data. Data was filtered for a minimum intensity of 1,000 and a sequence length between 6 and 30 with a maximum retention time RSD of 5%. Deuterium uptake was calculated, and HDX comparability profiles were plotted using an algorithm developed by Houde et al. (2011) incorporated into DynamX software.

#### Determination of Neonatal Fc Receptor-Anti-IL8 Binding Affinity

Affinity of produced anti-IL8 IgG1 antibody and neonatal Fc receptor (FcRn) was assessed based on surface plasmon resonance (SPR) biosensor binding assays conducted on a Biacore T100 instrument (GE Healthcare, Uppsala, Sweden) using Biacore T100 Control Software version 2.0.4. Reagents were all purchased from GE Healthcare unless stated otherwise.

Recombinant human FcRn (R&D systems, Minnesota, United States) was immobilized on a CM5 sensor chip using standard amine coupling. Thus, the dextran surface of two flow cells was activated by injecting a 1:1 solution of 1-ethyl-3-(3-dimethylaminopropyl)carbodimide hydrochloride and N-hydroxysuccinimide for 7 min at 10.0 μL/min. FcRn was diluted to 2.50 μg/mL in 10.0 mM sodium acetate pH 5.0 and injected at a flow rate of 5.0 μL/min onto the test flow cell until a response level of 500 RU was reached. Then, the surface of both cells was blocked by injecting 1.0 M ethanolamine pH 8.5 for 7 min at 10 μL/min. 50.0 mM sodium phosphate and 150.0 mM sodium chloride (NaCl, Sigma Aldrich), pH 6.0 at a flow rate of 30.0 μL/min was used as running buffer. Each mAb sample was injected in triplicate at the following concentrations: 0.0, 10.0, 25.0, 50.0, 100.0, 150.0, and 200.0 μg/mL. Injection was done at 25°C in a multi-cycle approach. Sensor chips were regenerated using 100.0 mM Tris and 200.0 mM NaCl, pH 8.0, for 1 min at 30.0 μL/min. Response was calculated at a position 4 s before the injection stop with a 5 s window. A steady state affinity fit model was then applied for determination of the dissociation constant using the Biacore Evaluation software (version 2.0.4.).

## Results

### Bioprocess Monitoring

Throughout the investigated bioprocesses, CHO cell growth, metabolism and recombinant protein production were monitored daily. During exponential phase of growth (day 1 to 5), all parameters measured followed a highly similar trend. When cells entered the stationary phase, conditions were changed, as outlined in Table 1, maintaining the central point of each experiment constant (37°C, 85% DO) to generate a biological triplicate that was used for reproducibility assessment. Figure 2 shows a comparison of biological replicates (standard conditions, gray). At the end of exponential phase on day 5 as well as before cell harvest on day 7, a very low experimental bias was observed. The relative standard deviation of the measured IgG concentration on day 7 was found to be below 1% (*n* = 3), highlighting the excellent repeatability that can be obtained using wave-mixed bioreactors. The biological triplicate obtained from standard conditions was also used for quality assessment of the proteomics data as discussed later.

High cell viability was maintained for the entire duration of batch culture (>85%). A decrease in temperature only resulted in a minor reduction of the growth rate while an increase to 39.5°C led to distinctly higher proliferation (Figure 2A). Increased proliferation at higher temperature did not result in increased lactate production whereas its concentration was found to be reduced to 31.9 mmol/L at 32°C. However, IgG concentration was more than 5.0 mg/L higher in the low temperature samples after 48 h of altered processing conditions. The productivity was found to be 2.29-fold increased at 32°C compared to standard conditions. An almost similar effect was observed when shifting the pH of the culture (Figure 2B), here, a 1.98-fold increase in productivity was observed. Increasing the pH to 7.2 caused increased growth along with higher lactate production (36.3 mmol/L). As shown in Figure 2C, changing the concentration of DO did not affect the viable cell density nor the productivity of the cells.

### Quantitative Proteomic Profiling Using LC/LC-SPS-MS^3^

After evaluating viability and productivity of CHO DP-12 cells, cell lysates were prepared for proteomic profiling to investigate the effects of altered bioprocessing conditions on endogenous protein expression and signaling pathways. Using high pH reversed phase offline fractionation followed by low pH reversed phase separation in combination with TMT labeling and SPS-MS^3^ detection, 7,385 CHO proteins (unique peptides ≥ 2) were identified, and 6,641 protein groups were successfully quantified (≤30% missing values). A full list of identified and quantified proteins can be found in Supplementary worksheet 1.

**FIGURE 3:**
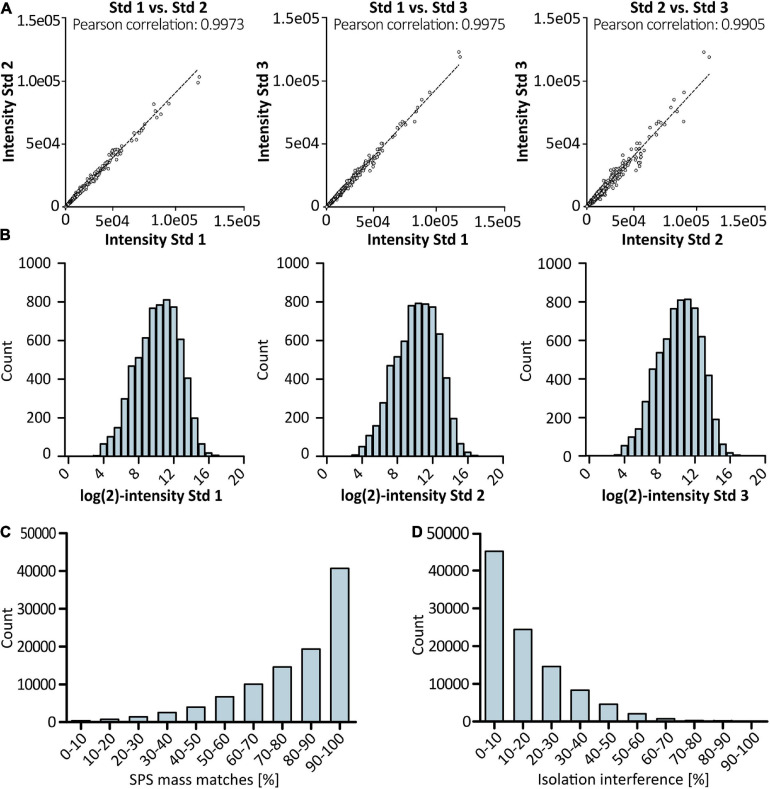
Results of proteomic profiling using LC/LC-SPS-MS^3^. Scatter plots in **(A)** show Pearson correlation between biological replicates derived from cells grown under standard conditions (*n* = 3). TMT reporter intensity values were log-transformed and plotted in a histogram **(B)** to show distribution of quantification values for control samples. Accuracy of quantification was evaluated by checking SPS match accuracy **(C)** and precursor ion co-isolation **(D)** in% of PSMs using Thermo Scientific’s Proteome Discoverer software. For each sample, two technical replicates were analyzed.

High reproducibility was observed for biological replicates derived from cells grown under standard conditions with a Pearson correlation coefficient of >0.99 (Figure 3A). Furthermore, the data follows the expected normal distribution as depicted in Figure 3B, with no indication for an under- or overestimation of protein intensities. Thermo Scientific’s Proteome Discoverer 2.3 enabled the evaluation of precursor selection and facilitated assessment of the accuracy of reporter ion quantification. More than 75% of peptide spectrum matches (PSMs) were found to have a SPS match accuracy of ≥70% (Figure 3C). Additionally, sample fractionation resulted in less than 30% of isolation interference (Figure 3D). Taken together, high SPS accuracy in combination with low isolation interference reduced potential effects of ratio compression and thus allowed for accurate quantitation.

**FIGURE 4:**
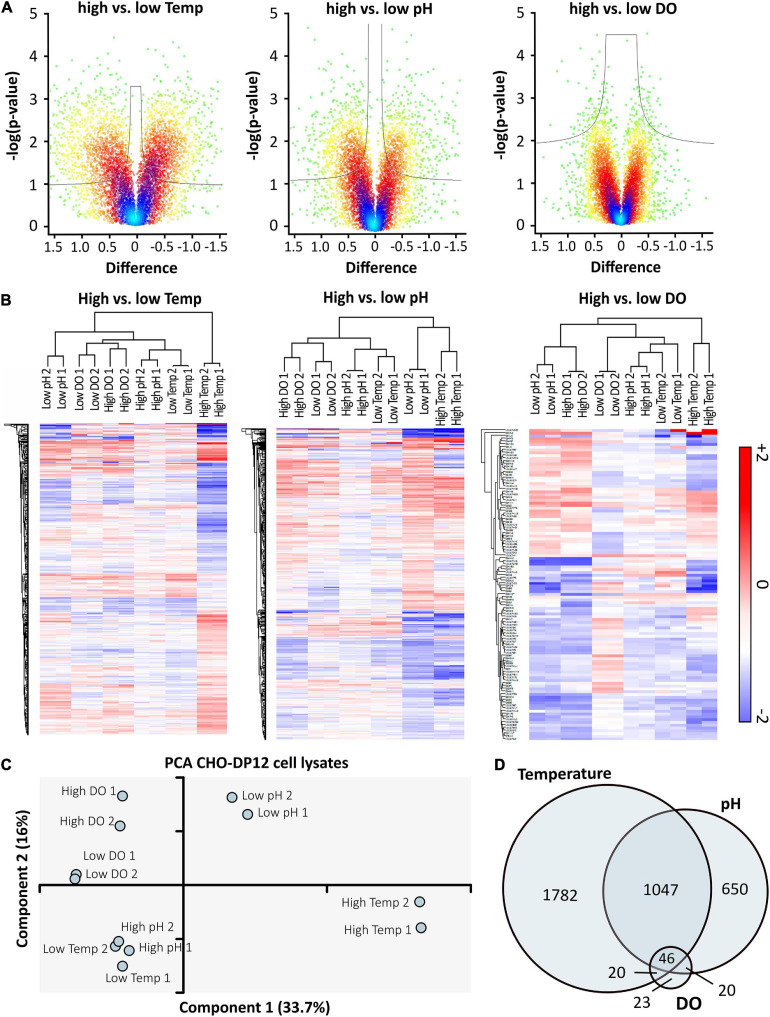
High vs. low cell culture conditions were compared using volcano plots **(A)** with cut-off curves indicating significance determined by a two-sided *t*-test with a permutation-based FDR of 0.05 and S0 of 0.03 (*n* = 2). Color of the plot indicates spatial density. Significantly regulated proteins were used for hierarchical clustering **(B)** based on Euclidian distance. Red indicates upregulation, blue shows downregulation of proteins compared to standard conditions. The PCA **(C)** indicates relationships between prepared technical replicates (*n* = 2) and applied processing conditions. The Venn-diagram **(D)** shows the overlap of significantly regulated proteins after shifting temperature, pH, or DO concentration. Data was analyzed using Perseus software (version 1.6.6).

To assess statistical significance of protein expression (Thompson et al., 2003), log-transformed ratios were compared (high vs. low) by means of a *t*-test using Volcano plots (Figure 4A). In line with the effects detected during bioprocess monitoring, the highest difference was observed when comparing the effect of high vs. low temperature shifts whereas changes in the DO concentration did not result in a strong effect on the level of protein expression. To further assess the quality of obtained data, hierarchical clustering was performed, showing clustering of prepared replicates, as depicted in Figure 4B. The heat maps only show significantly regulated proteins for the corresponding cell culture conditions. A similar effect was observed in the principal component analysis (PCA) shown in Figure 4C. In general, technical replicates showed good similarity, which is also depicted in Supplementary Figure 1. However, while different DO concentrations did not seem to have a large effect, high and low temperature as well as pH were found to be clearly separated. In accordance with results obtained from hierarchical clustering, a connection between cells grown at high pH and low temperature was observed. However, importantly, the limited overlap of significantly regulated proteins (Venn diagram, Figure 4D) implies that the applied conditions were affecting protein expression in different ways.

**FIGURE 5:**
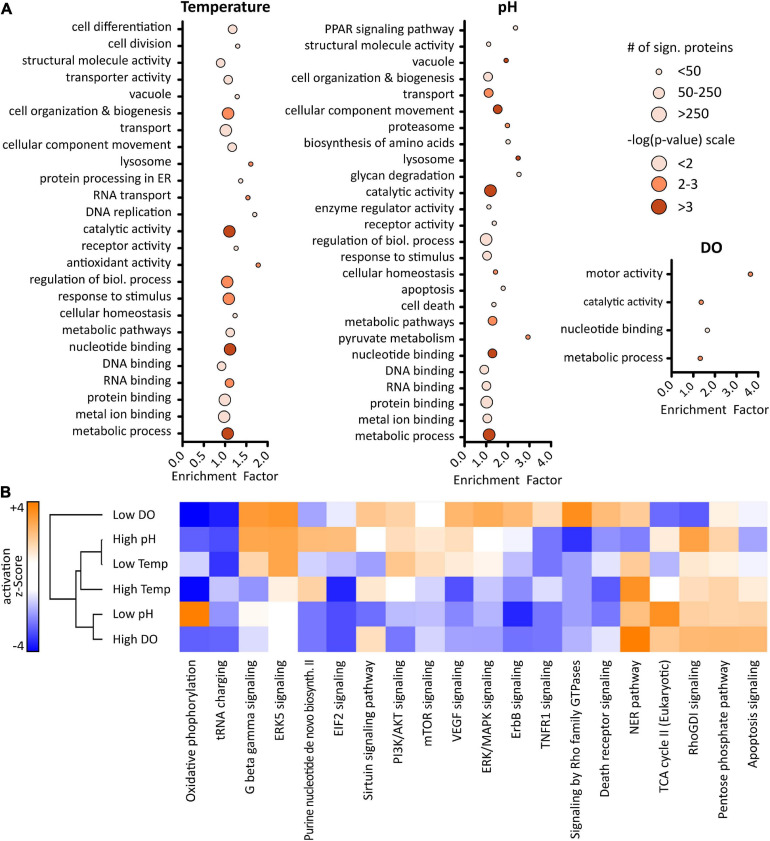
Results of gene ontology (GO) enrichment analysis **(A)** using a Fisher’s exact test with a *p*-value ≥ 0.05 performed in Perseus (version 1.6.6). Size of the dots represents number of proteins related to corresponding GO term, color indicates level of significance based on *p*-value. Ingenuity pathway analysis **(B)** revealed predicted pathway activation (orange) or inhibition (blue). Activation *z*-scores greater ± 2 are significant.

To enable biological insight, GO enrichment analysis was performed using a Fisher’s exact test with a *p*-value threshold of 0.05 in Perseus. 103 GO terms, composed of biological processes (GOBP), molecular functions (GOMF), cellular components (GOCC), and KEGG pathways, were found to be enriched in the dataset (Supplementary worksheet 2). Optimizing bioprocessing conditions generally focuses on increased culture viability and productivity. Therefore, attention was placed on associated GO terms like cell proliferation and growth, protein processing and transport, metabolism, and cell death or apoptosis. Results that could be related to these categories are shown in Figure 5A. Generally, varying culture conditions resulted in significant up- or down-regulation of proteins related to fundamental processes such as cell organization and biogenesis, cellular homeostasis, as well as metabolism.

Altered temperature was seen to influence proteins with antioxidant activity as well as protein processing in the ER. Additionally, while changing the temperature resulted in regulation of proteins related to cell differentiation and division, pH shifts appeared to influence apoptosis and cell death. Interestingly, changing the pH affected pyruvate metabolism and proteins with catalytic activity. Several proteins such as PDHA1, DLAT, and ME2, which are associated with pyruvate metabolism, were found to be up-regulated when reducing the pH of the cell culture media, while they were down-regulated at low temperature. PDHA1 and DLAT catalyze the conversion of pyruvate into acetyl-CoA, thus reducing the overall level of pyruvate present, which might be the reason for the lower lactate level that was observed. Even though a metabolomics experiment would be necessary for verification, these results suggest a switch in the metabolism leading at low pH to increased energy rather than lactate-production. Taken together, results from GO enrichment analysis indicate that altered bioprocessing conditions induce differential effects in IgG1 producing CHO DP-12 cells.

Whether a certain condition results in activation or inhibition of specific processes does not become clear solely by enrichment analysis. Therefore, gene names and log-ratios (altered condition vs. control) were used for pathway analysis in QIAGEN’s IPA using a mouse knowledge database. A complete list of canonical pathways can be found in Supplementary worksheet 3. Figure 5B shows activation or inhibition of the top 20 canonical pathways. *Z*-scores greater ± 2 are considered significant (Kramer et al., 2014). Since higher IgG concentration was observed after lowering the cell culture temperature or pH, a focus was placed on the effects induced by those conditions in order to obtain a deeper understanding of the underlying cellular mechanisms of recombinant protein production.

Generally, involvement of central pathways such as PI3K/AKT, mTOR, and ERK/MAPK which regulate important cellular mechanisms such as signal transduction, proliferation, or apoptosis was found. Those pathways were mostly activated upon lowering the temperature while, oppositely, being inhibited at low pH. Activation of PI3K/AKT signaling at low temperature, which plays an important role in cell cycle control, might be explained by higher expression levels of SHC1 and HRAS. Especially SHC1, which is important for the activation of RAS signaling, was found significantly up-regulated at low temperature whereas it was down-regulated at low pH. Furthermore, in line with the high viability observed throughout the whole cell culture, death receptor signaling, as well as TNFR1 signaling, were mostly found to be inhibited.

Most interestingly, lowering the pH resulted in a significant activation of oxidative phosphorylation whereas this pathway was inhibited in the other conditions. Additionally, pointing out the differential effects of the applied cell culture conditions, highly significant activation of the TCA cycle was observed in this condition which correlates well with the observed expression level of proteins. These results indicate that higher IgG production, upon lower cell culture temperature or pH, is based on different underlying biological processes.

In general, the combination of SPS-MS^3^ detection with prior high pH fractionation resulted in high proteome coverage with excellent reproducibility. Due to the altered processing conditions applied in the stationary phase of CHO cell growth, significant regulation of proteins involved in various biological processes and pathways relevant for process optimization was observed. Several of these observations are examined in the discussion.

### Characterization of Monoclonal Antibodies Following Production Under Altered Conditions

As the relationship between CHO cell signaling and mAb product quality is still poorly understood, complementary antibody characterization is crucial to fully evaluate the effects of bioprocessing conditions. Charge-related heterogeneity of the produced IgG1 was evaluated using strong cation exchange chromatography with UV detection (SCX-UV).

**FIGURE 6:**
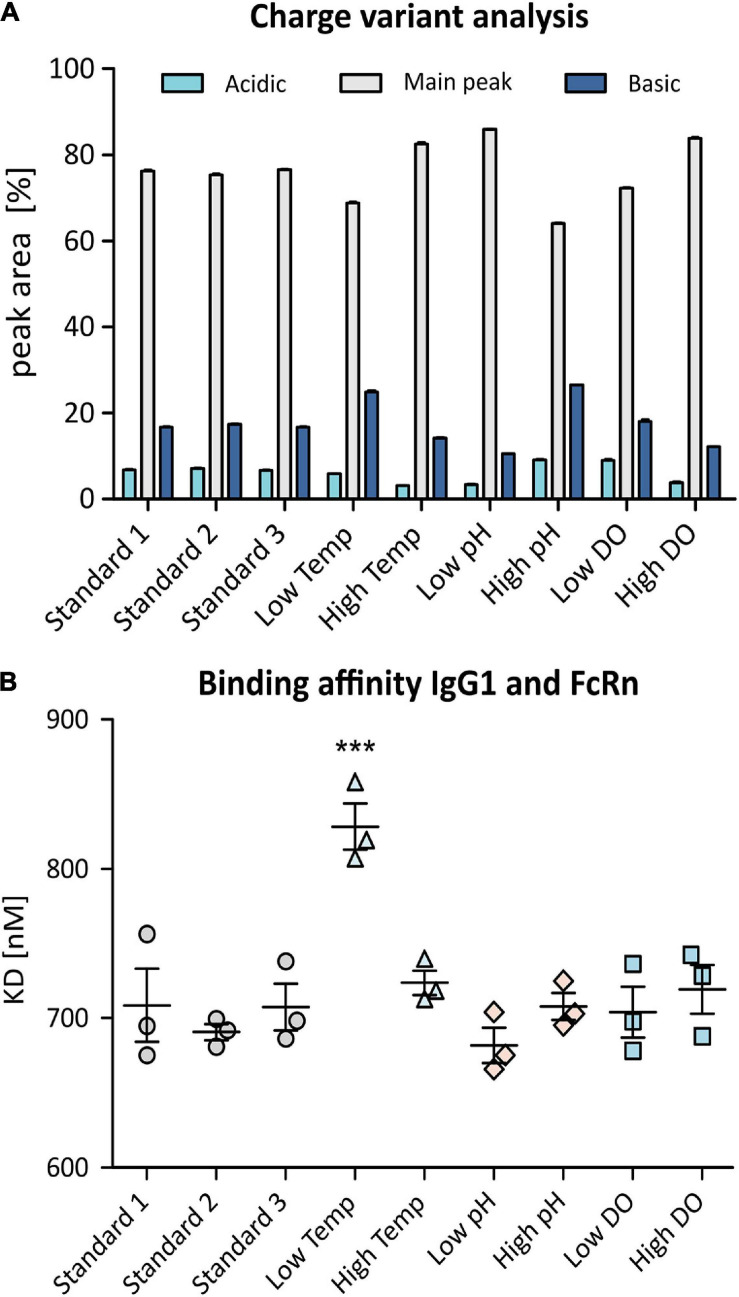
Charge variant analysis (CVA) of produced anti-IL8 IgG1 using SCX-UV **(A)**. Shown are relative peak areas [%] of the main peak (gray) as well as acidic variants (light blue) and basic variants (dark blue). SPR analysis was used to determine binding affinity of the mAb to recombinant human FcRn **(B)**. Samples were analyzed in triplicates. Shown are measured dissociation constants (KD). Significance was determined using an ANOVA with a Tukey’s post-test (***p ≤ 0.001).

This analysis resulted in the detection of a number of distinct peaks in addition to the antibody main peak which could be related to at least 7 charge variants present in each sample. Relative peak areas corresponding to collective acidic, basic, and main variants are shown in the bar chart in Figure 6A. While biological replicates obtained from standard conditions align well, an increase in basic variants was observed when lowering the temperature whereas higher temperature and high DO as well as low pH resulted in a reduction of basic and acidic variants. Increasing the pH of the batch culture increased the amount of both, acidic and basic variants of the mAb. Lowering the DO did not seem to influence the observed charge variant profile.

In order to characterize the post-translational modifications (PTMs) that are causing changes in the charge variant pattern, LC-MS based peptide mapping was performed. For each experimental condition, a sequence coverage of 100% was obtained. All modifications mapped on both, light and heavy chains, are shown in Supplementary worksheet 4.

The most prominent modification of the heavy chain was found to be *C*-terminal lysine truncation as well as asparagine deamidation, which are known to be causing the majority of acidic and basic variants that have been observed in CVA (Khawli et al., 2010). IgG1 obtained from cells grown at lower temperature or higher pH, showed lower levels of lysine truncation which corresponds with higher levels of basic variants. The applied experimental conditions also resulted in variable deamidation of N104 of the heavy chain, especially when comparing high vs. low temperature samples. Additionally, low levels of methionine and tryptophan oxidation were observed for all samples. Even though oxidation might not result in charge variants, it is noteworthy that a higher concentration of DO resulted in slightly higher levels of oxidation of the analyzed mAb.

Finally, structural and functional analysis of the produced mAb was performed using HDX-MS and SPR, respectively. HDX-MS can be used for comparative structural evaluation by determining changes in the mass of peptides in samples exposed to deuterated buffers. Higher levels of deuterium exchange point to amino acid accessibility due to potential structural changes. Differences in the deuterium uptake were determined with a set significance threshold of ±3 standard deviations (SD). As a result, 7 peptides were found to display significantly altered deuterium uptake, especially at low temperature but also when changing the DO concentration (high and low) as well as at high pH (Table 2). Interestingly, 3 of those 7 peptides were previously found to be oxidized or deamidated even though at low levels. Moreover, for samples obtained from low temperature batch culture, one peptide (T416-M433) with altered deuterium uptake was found to be in the Fc region of the antibody and another one (H39-I53) was found to be part of a complementarity-determining region.

**TABLE 2:** Peptides displaying altered deuterium uptake compared to standard mAb samples analyzed by HDX-MS and related peptide modifications identified by peptide mapping.

*Sample*	*mAb Chain*	*Peptide sequence*	*Position*	*Peptide modification*
*Low Temp*	Light	HWYQQKPGKAPKLLI	H39-I53	Oxidation W40
*High pH*	Light	AKVQWKVDNALQSGNSQE	A149-E166	Oxidation W153, Deamidation N157, Deamidation Q160, Deamidation Q163
*High DO*	Heavy	VQSGGGLVQPGGS	V5-S17	n/a
*Low DO*	Heavy	TLSRDNSKNTAY	T69-Y80	Deamidation N74, Deamidation N77
*Low Temp*	Heavy	VSWNSGALTSGVHTFPAVL	V161-L179	n/a
*Low Temp*	Heavy	NSGALTSGVHTFPAVL	N164-L179	n/a
*Low Temp*	Heavy	TVDKSRWQQGNVFSCSVM	T416-M433	n/a

Since differences in the deuterium uptake are an indication of changes in the higher order protein structure which could ultimately have an effect on safety, potency and efficacy of the mAb, functional analysis of the antibody is very important. Therefore, SPR was used to determine FcRn binding affinity which affects the mAb’s serum half-life and hence it’s efficacy (Roopenian et al., 2003; Mackness et al., 2019). For multi-cycle affinity SPR analysis, human recombinant FcRn was immobilized onto Biacore CM5 sensor chips before passing over increasing concentrations of anti-IL8 IgG1 to determine dissociation constants (KD) for the produced IgG1. As shown in Figure 6B, the binding affinity of the antibody and FcRn remained constant for most bioprocessing conditions. However, mAb production at lower temperature resulted in highly significant (*p* ≤ 0.001) reduction of binding affinity to FcRn which indicates a reduced serum half-life and thus an altered quality of the drug product.

## Discussion

In the present study, anti-IL8 IgG1 producing CHO DP-12 cells were cultured using shifts in temperature, pH and DO concentration, respectively. Applied changes in the DO concentration did not seem to have a strong impact on monitored parameters during cell culture which corresponds to previous findings (Restelli et al., 2006). However, wave bags used in this study, enable homogenous oxygenation of the cell culture. Still, DO mixing was shown to be problematic in large scale bioreactors, causing oxidative stress and thus lower productivity (Gao et al., 2016). As expected, the commonly applied decrease in temperature (Zou et al., 2018; Hennicke et al., 2019; McHugh et al., 2020) resulted in a more than two times higher IgG productivity and reduced cell proliferation as well as lower lactate production which also has been shown to impact productivity of the cells (Chen et al., 2001). An increased productivity, even though less pronounced, was also observed when lowering the pH of the culture.

Both lower temperature and lower pH resulted in increased productivity, although the product quality was observed to be different as discussed below. Therefore, special attention was placed on both of these conditions in order to examine the underlying cellular mechanisms. While there is evidence that reduced temperature enhances productivity *via* induction of a cell cycle arrest in CHO cells (Becerra et al., 2012), it seems that the lower cell culture pH is increasing recombinant protein expression by enhancing the cellular energy metabolism (Becker et al., 2019). In order to expand the current knowledge about cellular effects of these commonly applied bioprocessing conditions, proteomic profiling of CHO cell batch cultures was performed.

Quantitative SPS-MS^3^ based LC-MS analysis yielded excellent reproducibility with improved proteome coverage (Bertrand et al., 2019; Lakshmanan et al., 2019) and accurate quantification of proteins (Figure 3) without the need of intense fractionation (Heffner et al., 2017). Statistical data analysis by means of PCA (Figure 4C) indicated that the cellular effects underlying an improved antibody expression at low temperature and low pH differ considerably. At the same time, only minor changes in the protein expression profile of cells grown at altered DO concentration were observed, corresponding to the results of the bioprocess monitoring.

### Recombinant Protein Production at Lower Cell Culture Temperature Is Increased Due to an Affected Cell Cycle

Gene ontology enrichment analysis (Figure 5A) revealed that altered temperature resulted in the enrichment of proteins related to cell differentiation and cell division. In relation to this, pathway analysis (Figure 5B) showed an activation of pathways regulating proliferation, e.g., PI3K/AKT and mTOR. In addition, enhanced ERK5 signaling together with an inhibition of the TNFR1 pathway in cells grown at reduced temperature implied inhibition of apoptosis (Nishimoto and Nishida, 2006). As summarized by Becerra et al., a decrease in culture temperature can improve productivity in multiple ways but it is mainly dependent on the cell line and recombinant protein. Even though there is no unified explanation for the increased productivity, most studies reported an induced cell cycle arrest which aligns well with results of our GO and pathway analysis.

### Improved mAb Productivity of Cells at Low pH Is Due to Improved Energy Metabolism

Most interestingly, reduction of the culture pH induced a highly significant enhancement of oxidative phosphorylation, as well as an activation of the TCA cycle in the CHO cells. This finding agrees well with the enrichment of proteins involved in pyruvate metabolism (Gray et al., 2014), namely PDHA1, DLAT, and ME2 and their increased expression levels. It was shown previously that improved ATP production at low pH (Ivarsson et al., 2015) and concurrent increased energy availability is improving the antibody production rate (Hartley et al., 2018; Becker et al., 2019). Additionally, it has been reported that cellular redox homeostasis is important to maintain high productivity. Interestingly, we found two proteins with antioxidant activity, PRDX5 and SOD2, to be up-regulated at low pH whereas, in contrast, this was not the case after culturing cells at low temperature. On the other hand, ATF6 was found to be significantly up-regulated after lowering the temperature while being down-regulated at low pH. ATF6 is a transcription factor which plays an important role in the cellular response to ER stress, especially in the unfolded protein response. Even though expression was not increased for the chaperone proteins measured, a significant upregulation of BAK1 and Caspase-3 was observed indicating ER stress. While it would need additional experiments to verify our findings on the level of protein expression, it seems that in both cases, lowering the pH has beneficial effects on maintaining redox balance which is ultimately favoring recombinant protein expression (Ali et al., 2019, 2020).

Generally, combining the proteome analysis with additional omics-data, such as metabolomics or phosphoproteomics, would be beneficial to allow for a deeper biological interpretation even though it increases the experimental effort and time. Still, proteome analysis of CHO DP-12 cells obtained in this work from altered bioprocessing conditions clearly pointed out the differences of how those conditions affect the cells and hence their productivity. Especially the effects of altered pH on the energy metabolism of the cells were shown.

### Cell Culture Conditions Affect Expression Levels of Carboxypeptidase and Therefore Lead to Differential Lysine Truncation

Following characterization of the produced mAb by CVA revealed altered levels of acidic and basic charge variants, particularly at low temperature (Figure 6A). This was related to altered *C*-terminal lysine clipping and differential deamidation of N104 as observed in subsequent peptide mapping. Both modifications are commonly seen in the production of mAbs and are known to affect the charge variant profile. While deamidation is usually induced during up- and downstream processing, lysine clipping has been shown to be depending on endogenous carboxypeptidase activity (Liu et al., 2016; Fussl et al., 2018). Interestingly, carboxypeptidase (Cp, G3H1D5) levels were found to be directly related with the observed abundance of lysine. A reduction of cell culture temperature also reduced Cp expression going along with higher amounts of *C*-terminal lysine while the opposite was the case at low pH. This shows that observed protein expression levels can directly be linked to the product quality profile.

### Structural and Functional Analysis of IgG1 Produced at Altered Bioprocessing Conditions Revealed Potentially Altered Efficacy of the Antibody

Hydrogen-Deuterium Exchange Mass Spectrometry revealed possible structural changes in 7 peptides of the produced mAb which could not be related to differences in the glycosylation (data not shown). Interestingly, reduced temperature resulted in altered deuterium uptake in the Fc region of the IgG1 which corresponded with a highly significant reduction of the affinity toward FcRn shown by SPR (Figure 6B). Importantly, the FcRn binding affinity of the produced mAb remained constant for the other experimental conditions, also at low pH. This suggests a reduced serum half-life (Roopenian et al., 2003) and therefore altered quality of the mAb obtained after shifting the temperature to 32°C. However, while it was previously reported that PTMs such as methionine oxidation or altered glycan composition can affect FcRn binding affinity (Houde et al., 2010), no modifications of this particular region were detected in this study. Also, while structural changes did not directly affect known FcRn binding sites (Shields et al., 2001; Pyzik et al., 2019), it is possible that changes in the higher order protein structure of the Fc part of the IgG1 under investigation are causing reduced affinity. This requires further study.

### Altered Cellular Metabolism Upon Differential Bioprocessing Affects Productivity as Well as Product Quality

In conclusion, applied bioprocessing conditions were found to differentially affect the metabolism of IgG1 producing CHO DP-12 cells. While decreased temperature as well as reduced pH resulted in increased recombinant protein production by the cells, different cellular mechanisms were found to be involved in this process. Additionally, reduced temperature seemed to have more intense effects on the quality of the produced antibody, altering the binding affinity to FcRn, which was not the case at low pH. Furthermore, differences in the observed charge variant profile could be directly linked to altered protein expression levels. Taken together, these observations allow for the conclusion that a higher recombinant protein production rate due to increased ATP availability does not detrimentally affect product quality. Whether this is only the case for the particular cell line and antibody under investigation, needs further examination. Nevertheless, the results demonstrate the importance of combining in-depth proteomic profiling with intense product characterization to enable molecular and biochemically driven improvements of manufacturing processes.

## Data Availability Statement

The datasets presented in this study can be found in online repositories. The names of the repository/repositories and accession number(s) can be found in the article/Supplementary Material.

## Author Contributions

AF and JB conceived the project. AF performed the majority of the experimental sections. JH, PP, KS, PM, and RV provided raw data for proteome analysis. Peptide mapping was done by AF and KS. LS analyzed proteomics data and drafted the manuscript. LS, AF, BK, and JB were involved in data interpretation. All authors reviewed the manuscript.

## Conflict of Interest

JH, KS, KC, RV, PP, and PM are employed by Thermo Fisher Scientific, the company that develops and produces the MAbPac SCX, Acclaim and Easy Spray Acclaim columns, Vanquish UHPLC system and Orbitrap mass spectrometers. The remaining authors declare that the research was conducted in the absence of any commercial or financial relationships that could be construed as a potential conflict of interest.

## Abbreviations

ACN: Acetonitrile
AGC: automatic gain control
CDR: complementarity-determining region
CHO: Chinese hamster ovary
CVA: charge variant analysis
DO: dissolved oxygen
DTT: dithiothreitol
FA: formic acid
FASP: filter-aided sample preparation
FcRn: neonatal Fc receptor
GO: gene ontology
GOBP: biological process
GOCC: cellular component
GOMF: molecular function
HDX-MS: Hydrogen-Deuterium Exchange Mass Spectrometry
IAA: iodoacetamide
iTRAQ: isobaric tags for relative and absolute quantitation
KD: dissociation constant
LC: liquid chromatography
mAb: monoclonal antibody
MS: mass spectrometry
MWCO: molecular weight cutoff
NCE: normalized collision energy
PBS: phosphate buffered saline
PCA: principal component analysis
PSM: peptide spectrum match
SCX: strong cation exchange
SPS: synchronous precursor selection
TCEP: tris(2-carboxyethyl)phosphine
TEAB: triethylammonium bicarbonate
TMT: tandem mass tag.
