# Supplementary material for: Proteomic Profiling of IgG1 Producing CHO Cells Using LC/LC-SPS-MS3: The Effects of Bioprocessing Conditions on Productivity and Product Quality
Source: Front Bioeng Biotechnol. 2021 Apr 9;9:569045. doi: 10.3389/fbioe.2021.569045 (PMC8062983; doi:10.3389/fbioe.2021.569045)
Supplement: Supplementary file 2 [file Data_Sheet_1.docx]

Supplementary Material

## Supplementary Figures


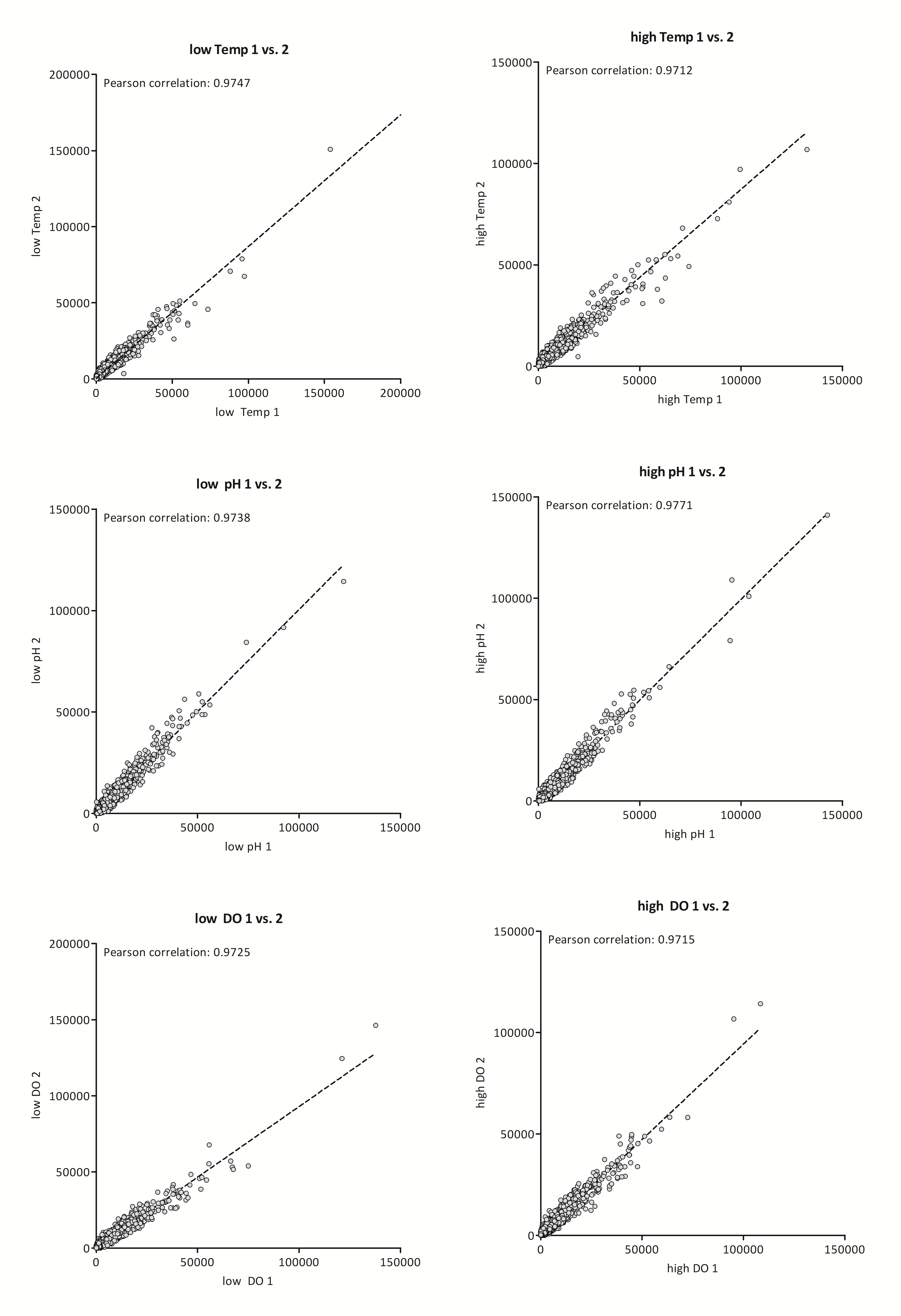


**Supplementary Figure 1.** Results of proteomic profiling using LC/LC-SPS-MS^3^. Scatter plots show Pearson correlation between replicates derived from cells grown under altered conditions (n=2).
